# The Relationship between Ultraviolet Radiation Exposure and Vitamin D Status

**DOI:** 10.3390/nu2050482

**Published:** 2010-05-04

**Authors:** Ola Engelsen

**Affiliations:** Norwegian Institute for Air Research (NILU), The Polar Environmental Centre, NO-9296 Tromsø, Norway; Email: ola.engelsen@nilu.no

**Keywords:** vitamin D, ultraviolet, UV, sun

## Abstract

This paper reviews the main factors influencing the synthesis of vitamin D, with particular focus on ultraviolet radiation exposure. On the global level, the main source of vitamin D is the sun. The effect of solar radiation on vitamin D synthesis depends to some extent on the initial vitamin D levels. At moderate to high latitudes, diet becomes an increasingly important source of vitamin D due to decreased solar intensity and cold temperatures, which discourage skin exposure. During the mid-winter season, these factors result in decreased solar radiation exposure, hindering extensively the synthesis of vitamin D in these populations.

## 1. Introduction

Vitamin D is an important nutrient for maintaining a healthy skeleton as it forms an integral part of the bone metabolism, calcium and phosphor homeostasis [1]. There are indications that vitamin D may have several other health benefits such as prevention or mitigation of cancer [2] and autoimmune diseases [3], reduction in hypertension [4], and prevention of influenza [5]. Anti-carcinogenic effects have been demonstrated in laboratory studies on animals at high doses, but evidence of causality has not been shown in humans, possibly due to too low vitamin D levels in the human population to produce a statistically significant effect [2]. Overall, vitamin D seems to have a positive regulatory effect on the immune system. Recent research indicates that vitamin D stimulates antimicrobial activity [6] and thus may mitigate certain types of infections. There are vitamin D receptors in many organs [7], and long-term vitamin D deficiency may induce a wide range of harmful biological effects. Despite this, only skeletal effects have to date been proven causal in a strict sense [8,9,10]. The existence of an optimal ultraviolet (UV) exposure and its level is a subject of ongoing debate [2,11,12,13,14,15]. Likewise, public recommendations on vitamin D dietary intake and food fortifications are under revision in many countries.

Worldwide studies have shown that vitamin D status is low across wide ranges of populations and age groups even at very moderate latitudes [16,17,18,19,20,21,22,23,24]. Extremely low vitamin D levels have been observed in individuals with darker skin [25,26,27]. It seems clear that casual vitamin D intake from UV exposure and diet are not adequate. Improved public consciousness in combination with revised policies regarding vitamin D can very likely improve public health at quite moderate costs.

**Figure 1:**
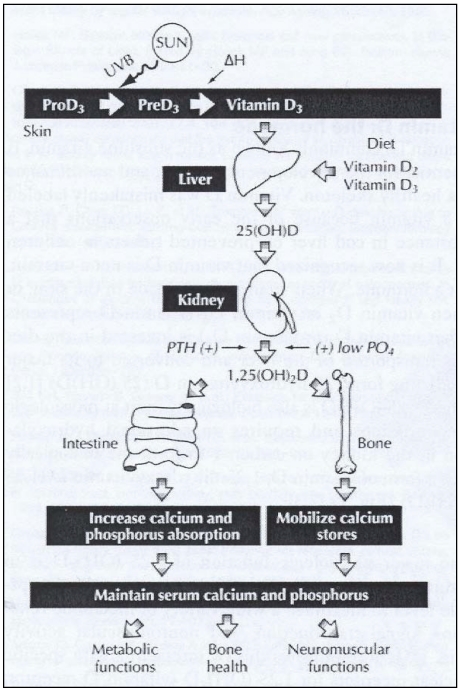
Outline of vitamin D synthesis and metabolism. From [7].

The formation, intake and circulation of vitamin D are well described in the literature, and are outlined in Figure 1. Provitamin D (7-dehydrocholesterol, 7-DHC) is converted to previtamin D in the skin by exposure to UVB radiation. The previtamin D is then isomerized by body heat to form vitamin D_3_. Vitamin D_3_ is then transported by the blood to the liver, where it is converted to 25-hydroxyvitamin D (25(OH)D). In the kidneys, the formation of the active form of vitamin D, 1,25-dihydroxyvitamin D (1,25(OH)_2_D), is tightly regulated by the parathyroid hormone (PTH). 1,25-dihydroxyvitamin D is important for the uptake of calcium and mobilization of calcium stores. As mentioned above, many organs have receptors for vitamin D, and consequently vitamin D is important for many bodily functions. More recently, it was found that 1,25-dihydroxyvitamin D can be produced in tissue and cells, decoupling partly the production of 1,25-dihydroxyvitamin D and its effects from the renal functions and PTH, reviewed in [28]. Although the underlying processes of vitamin D synthesis and circulation is known, the quantitative relationship of UV exposure to humans and the resulting vitamin D production is inadequately described. This review summarizes the status of knowledge with respect to vitamin D synthesis in the human skin, and attempts to clarify the gaps of knowledge as an aid to future research in the area.

## 2. Ultraviolet Radiation Exposure and Effects on Cutaneous Vitamin D Synthesis

The fundamental processes of vitamin D synthesis and circulation in humans is well understood at a qualitative level (Figure 1). *In vitro* models [29,30,31] and experiments [32,33,34] describing photoconversion of provitamin D to previtamin D exist. Although they are not necessarily representative for the cutaneous and systemic conditions on a large human population level, they are useful qualitative indicators for how much vitamin D is produced under various solar conditions. 

**Figure 2:**
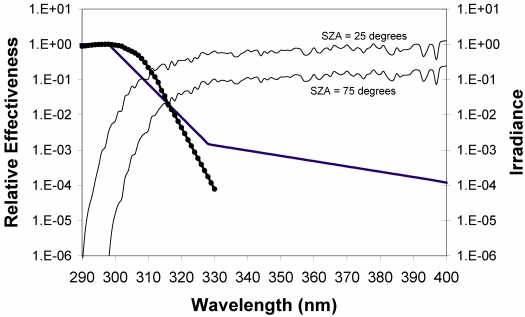
CIE erythema action spectrum [60] (bold line) and the action spectrum for the formation of previtamin D in human skin [37 ](dots) with solar spectra (thin solid lines) measured at solar zenith angles of 25 and 75 degrees. The units of the irradiances in the solar spectra are Wm^-2^nm^-1^. From [15], and the relative effectiveness of the action spectra are unitless.

The factors affecting vitamin D synthesis from ultraviolet B (UVB) radiation have been discussed previously [35,36]. The effect of some influencing factors can be further explored by running the online facilities described in section 5 for user-specified conditions. The factors are therefore only discussed briefly below:

- Solar zenith angle (season and latitude) have a substantial impact on UVB radiation. At low solar zenith angles, photons must travel longer distances through the ozone layer, increasing the probability of absorption. There is also an enhanced possibility of interaction with air molecules, leading to absorption or scattering back into space, thus effectively attenuating UV radiation (Figure 2). Because the atmosphere attenuates UV radiation differently for various wavelengths, the UV spectrum varies with solar elevations. The vitamin D effective radiation is described in terms of its action spectrum (*i.e.,* the efficiency of each wavelength to synthesize vitamin D in skin) [37]. In broad terms, the action spectrum covers the UVB spectral range with a maximum at about 295 nm (Figure 2).
- Clouds can both attenuate and enhance UVB radiation, although attenuation is generally the case. Completely overcast clouds always attenuate UVB rays, even up to 99% of UVB radiation in extreme cases [38]. Up to 50% enhancement of UVB radiation can occur from broken clouds [39] or at elevated sites above clouds [40].
- Ozone effectively absorbs UVB radiation, particularly at shorter wavelengths [41]. Besides clouds, it is the most important atmospheric modulator of vitamin D synthesis.
- Surface reflection, from snow in particular, reflects up to 95% of UVB radiation [42].
- Altitude. Solar UVB radiation increases by about 7% every km in altitude under clear sky conditions, and more if the subject is in or above clouds or a turbid atmosphere.
- Sunscreen blocks UVB radiation effectively [43,44]. However, it is questionable whether sunscreen in practise causes any vitamin D deficiency. Absolute full-body coverage of sunscreen is uncommon. Some areas of the skin are always left out. At times and locations where the sun is intense and the temperature is high enough to make the population use sunscreen, its vitamin D status is generally very satisfactory.
- Outdoor behavior. There is an ongoing trend towards less outdoor exposure, either through work or preferences in leisure activities. For instance, children in the USA now only spend half-an-hour outdoors a day during week-ends, and only minutes during week-days [45]. Furthermore, the orientation of the skin with respect to the sun has a great impact on the personal UV exposure. Nearby objects can obstruct both direct and diffuse UV rays [46], and thus affect UV synthesis. The best way to obtain precise personal UV exposure is dosimeters [47].
- Skin type. Dark (type VI, [48]) skins produce up to six-times less vitamin D than pale (type I) skins [49,50,51,52,53,54].
- Obesity. Overweight individuals have reduced capacity of vitamin D synthesis [55].
- Age. Elderly people have thinner skin, and consequently are less capable of synthesizing vitamin D in their skin [56].
- Sun beds. The use of sun beds is controversial, but regardless, subjects who regularly use tanning beds that emit UVB radiation are likely to have higher 25(OH)D concentrations [20] and also higher bone mineral densities [57].
- Clothing (temperature). At cold temperatures the population wears more clothes for comfort, exposing less skin area to UVB radiation, and thereby inhibiting vitamin D synthesis [58,59]. At moderate and high latitudes, face, neck and hands are generally exposed at best. During freezing temperatures, only the face is usually exposed.

## 3. Comparative Effect of Diet and UV Exposure on Serum 25-hydroxyvitamin D (25(OH)D) Level

Holick and co-workers found that one full body UV exposure causing a slight pinkness in skin (one minimum erythemal dose, 1 MED) is equivalent to an oral intake of somewhere in the range 250–625 μg (10,000–25,000 IU) of vitamin D_3_ [61,62,63]. This relationship has been confirmed by at least three other studies (see below), including outdoor solar exposure and on only parts of the body. A weakness of the laboratory experiments lies in the fact that the experiments made use of fixed broadband UV lamps. The lamp spectral characteristics are unclear, and assimilation to natural conditions is difficult due to uncertainties in vitamin D action spectrum. Furthermore, relevant laboratory experiments are generally done for very small cohorts in the literature, and this was also the case for these studies. On the basis of the results of Holick and co-workers above [64,65], a UV exposure of one-quarter of personal MED on one-quarter of skin area (hands, face and arms) yields a dietary equivalent vitamin D dose of about 1000 IU. The exposure times to obtain this recommended UV dose depend greatly on skin type, time and location [65] as well as ambient conditions and clothing [53]. The latter paper has resulted in a freely accessible internet page, which gives an estimate of how long exposure is required to obtain any vitamin D-effective dose from the sun under user-specified conditions (http://nadir.nilu.no/~olaeng/fastrt/VitD_quartMED.html). Limitations of the method have been previously discussed [53]. Beneficial exposure times for Australian cities have also been computed from measurements of UV erythemal doses [66]. They assume that one-third to one-sixth MEDs at 15% of the body yield the equivalent of the current officially recommended vitamin D dose of 200-600 IUs. Note that the relationship between erythemally weighted doses (or MEDs) and vitamin D effective doses with respect to solar zenith angle is not linear [67,68]. Therefore, the amount of MEDs can be misleading in terms of cutaneous vitamin D synthesis.

As mentioned above, at least three additional studies support the concept that one full-body exposure to sunlight can be equivalent to an oral vitamin D intake of 250 μg (10,000 IU) [69]. “Stamp [70] compared oral vitamin D to the effects of UV light treatment sessions (~1 MED) and found that the rise in 25(OH)D was the same in subjects treated with UV light as in those given 250 μg (10,000 IU) vitamin D/d. In a study of institutionalized elderly, Davie *et al.* [71] exposed 600 cm^2^, ~5% of skin surface, to UV light treatments over a 2–3-month period and compared the resulting 25(OH)D concentrations with those achieved with oral vitamin D doses. They calculated a production of vitamin D in the skin equivalent to 0.045 nmol・d^-1^・cm^-2^ exposed skin. This is equivalent to 10.9 μg (435 IU) vitamin D/d for 600 cm^2^ (5%) of skin surface. If these results for the elderly are extrapolated to total body surface area, it works out to 218 μg (8,700 IU) vitamin D/d that can be acquired by the elderly.” Chel *et al.* [72] obtained very similar results for a half MED exposure of 1000cm^2^ (one-sixth of the body area) yielding the equivalent of 400 IU corresponding to 9,600 IU for a full body MED exposure. Note that it is well established that the elderly are less capable of synthesizing vitamin D in their skin (see above). Also, their starting 25-hydroxyvitamin D levels were generally quite low (25(OH)D < 25 nmol/L in Holick [61], patients requiring vitamin D treatments in Stamp [70], 25(OH)D~5.5–16.6 nmol/L in Davie *et al.* [71]. 25(OH)D < 30 nmol/L for Chel *et al.* [72]). Low vitamin D status yields higher sensitivity to vitamin D intake than normal [see below]. Furthermore, no vitamin D effective doses are given, only the number of MEDs is given as an approximate measure of UV radiation dose.

There are several studies on the separate effects of dietary intake and UVR exposure on serum 25-hydroxyvitamin D levels. However, there are markedly less studies on the effects on UVR exposure, probably due to a more complex experimental setup as well as ethical problems by exposing subjects with knowingly harmful UV radiation. An extensive list is given in tables 2 and 4 of Vieth [69] and Vieth [73]. Other studies include [33,74,75,76,77]. Davie [71] devised empirical formulas describing the effect of both dietary intake and UV radiation on the serum 25-hydroxyvitamin D levels based on results from 25 subjects. Devising exact, general formulas are not straightforward because the effect of vitamin D intake on vitamin D production depends on the initial 25(OH)D level. It seems that the effect of vitamin D intake and synthesis is enhanced when the initial vitamin D status is low [69,78,79]. A problem in making comparisons between *in vivo* studies on the effect of UVR exposure is that evaluation of the exact UV doses is complicated by variations in the surface area of skin exposed, skin type, starting levels of serum 25-hydroxyvitamin D, and frequency and duration of exposure. Furthermore, the spectra of the radiation sources are usually variable and inadequately specified, and computing comparable vitamin-D effective doses from the given information is not straightforward. Exact assimilation of the various studies is thus difficult.

Several journal papers have described the statistical relationships between vitamin D status and an incomplete set of indirect parameters (latitude, hours of exposure, *etc.).* Unfortunately, many of those extensive cohort studies do not contain adequate information to reconstruct the relevant UVR doses at a desirable accuracy.

**Figure 3:**
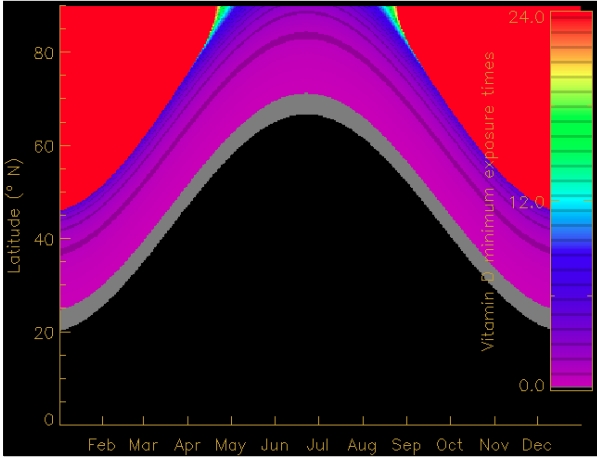
Required UV exposure times around noon for a cloudless sky, and typical conditions with respect to latitude and day of year to obtain approximately the equivalent of 400 IU when the face, neck and hands with type I skin are exposed. The red areas illustrate when this vitamin amount is not achievable from the sun. The required dose can be obtained in minutes in the black area. From [36,53].

It has been often claimed that adequate vitamin D is obtained after a few minutes of sun exposure. This is commonly true only when the sun is high and if the equivalent of 400 IU (corresponding to one spoonful of cod liver oil) is desired (Table 1, Figure 3). However, many research scientists advocate higher intake levels of vitamin D (1,000–4,000 IU). These amounts are usually not available from casual daily exposure (e.g., waiting for the bus to work). The gap between beneficial UV exposure to obtain desirable vitamin D and harmful exposure leading to erythema also narrows (*i.e.,* desirable vitamin D effective dose approaches 1 MED [15]). For example, for normal summer clothing, the exposed skin (25.5%) needs to be sunburnt in order to produce 4,000 IU. The skin would then receive a UV dose associated with clearly elevated skin cancer risk.

**Table 1:** Exposure time in hours and associated MED (in parentheses) for Boston at the spring equinox for all permutations of the variables dietary intake (IU), Skin type (2, 5) and skin area of exposure (F-face, N-neck, H-hands, A-arms, L-legs). From [15].

Vit. D >	400 IU		1000 IU		4000 IU	
**Skin Type>**	**2**	**5**	**2**	**5**	**2**	**5**
**Area**						
**F,N,H (11.5%)**	0.15 (0.21)	0.35 (0.21)	0.36 (0.54)	0.89 (0.54)	1.49 (2.16)	3.95 (2.16)
**F,N,H,A (25.5%)**	0.07 (0.09)	0.16 (0.09)	0.17 (0.24)	0.40 (0.24)	0.67 (0.97)	1.62 (0.97)
**F,N,H,A,L (57.5%)**	0.03 (0.04)	0.07 (0.04)	0.07 (0.10)	0.18 (0.10)	0.29 (0.43)	0.70 (0.43)

## 4. Seasonal and Latitudinal Effects on Vitamin D Synthesis and the Vitamin D Winter

**Figure 4:**
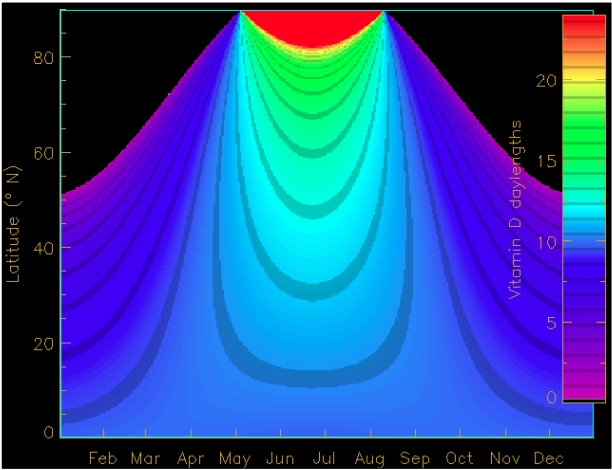
Daily period (in hours) of vitamin D production in terms of time and latitude for a typical clear atmosphere and no surface reflection. From [83].

A dramatic effect of seasonal and latitudinal changes of solar UV radiation on vitamin D synthesis was revealed by Webb *et al.* [80]. This work showed that from November to February, there was insufficient solar UVB to synthesize vitamin D in Boston, MA (USA), but by March, previtamin D was formed from 7-DHC in both solution and the skin. This observation, also known as the “vitamin D winter”, has been reported in other journal studies [81,82]. More moderate estimates of the extent of this “vitamin D winter” have been obtained; however, are based on simulations of UV radiation for clear atmospheric conditions (Figure 4) [83]. The vitamin D effective UV doses depend significantly on latitude and season [84,85]. Vitamin D production is generally seasonally dependent, being low during late winter with exception to some high-latitude coastal communities where diet seems to be the dominant source of vitamin D [86]. There seems to be slightly lower vitamin D levels in women probably due to more body fat [87]. Latitude, location, and time of year may also have health implications in regards to vitamin D, where several studies have associated development of various diseases, influenced by vitamin D deficiency, with geography and season, e.g. [88,89].

## 5. A Note on Online Vitamin D Calculator Facilities

User-operated model simulations for vitamin D synthesis and effective doses are available online:

1. Vitamin D winter duration [83] (http://nadir.nilu.no/~olaeng/fastrt/VitD.html).
2. UV exposure times to substitute dietary intake [53] (http://nadir.nilu.no/~olaeng/fastrt/VitD_quartMEDandMED.html).
3. Vitamin D effective doses [85] (http://nadir.nilu.no/~olaeng/fastrt/fastrt.html).

The user can insert their own atmospheric and surface conditions and calculate UV doses and exposure times for arbitrary times and locations. Note that the results from three online model simulations are not entirely consistent. This is due to the laboratory experiments forming the basis for the calculations of the online facilities. Tool 1 is based on comparison to *in vitro* experiments for low intensities of UV radiation. Tool 2 on the other hand is based on extrapolations from intense erythemal doses of young adults.

## 6. Conclusions and Suggestions for Additional Research

The combined effects of UV radiation and diet on vitamin D status should be explored more rigorously, both in laboratory environments and at the population level.

- There is a good qualitative understanding of underlying processes, but still cutaneous UV synthesis is inadequately understood for practical purposes. Quantitative modeling is possible, but it is incomplete and is based on very limited cohort experiments.
- Individuals risk sun burn if high doses of vitamin D should be obtained by normal skin exposure (face, neck, hands).
- Unrealistically long exposure times are sometimes required to obtain recommended vitamin D doses through skin.
- Desirable vitamin D doses and erythemal doses are more similar for low solar elevations.

## Notes Added in Proof

In the first paragraph of section 3 I state that "The lamp spectral characteristics are unclear, and assimilation to natural conditions is difficult due to uncertainties in vitamin D action spectrum." Regarding the lab experiments of Holick and coworkers, this has now been clarified in John C. Dowdy, Robert M. Sayre, Michael F. Holick, Holick's rule and vitamin D from sunlight, The Journal of Steroid Biochemistry and Molecular Biology, In Press, doi:10.1016/j.jsbmb.2010.04.002. These results will improve simulation model #2 in section 5.
